# Comparative cytogenetic of six species of Amazonian Peacock bass (*Cichla*, Cichlinae): intrachromosomal variations and genetic introgression among sympatric species

**DOI:** 10.3897/CompCytogen.v14i3.55279

**Published:** 2020-09-17

**Authors:** Janice Quadros, Alex M. V. Ferreira, Patrik F. Viana, Leandro Marajó, Ezequiel Oliveira, Efrem Ferreira, Eliana Feldberg

**Affiliations:** 1 Laboratório de Genética Animal, Coordenação de Biodiversidade, Instituto Nacional de Pesquisas da Amazônia, Av. André Araújo 2936, Petrópolis, 69067-375, Manaus, AM, Brazil; 2 Laboratório de Ecologia de peixes, Coordenação de Biodiversidade, Instituto Nacional de Pesquisas da Amazônia, Av. André Araújo 2936, Petrópolis, 69067-375, Manaus, AM, Brazil; 3 Departamento de Genética e Evolução, Universidade Federal de São Carlos, São Carlos, São Paulo 13565-905, Brazil

**Keywords:** 5S rDNA, FISH, Heterochromatin, Hybridization, karyotype

## Abstract

Cytogenetic data for the genus *Cichla* Bloch et Schneider, 1801 are still very limited, with only four karyotype descriptions to date. The sum of the available cytogenetic information for *Cichla* species, points to a maintenance of the diploid number of 48 acrocentric chromosomes, considered a typical ancestral feature in cichlids. In the current study, we performed molecular and classical cytogenetic analyses of the karyotype organization of six species of *Cichla*, the earliest-diverging genus of Neotropical cichlids. We cytogenetically analysed *Cichla kelberi* Kullander et Ferreira, 2006, *Cichla monoculus* Agassiz, 1831, *Cichla piquiti* Kullander et Ferreira, 2006, *Cichla temensis* Humboldt, 1821, *Cichla vazzoleri* Kullander et Ferreira, 2006 and *Cichla pinima* Kullander et Ferreira, 2006, including three individuals that showed mixed morphological characteristics, likely from different species, suggesting they were hybrid individuals. All individuals analysed showed 2n = 48 acrocentric chromosomes, with centromeric heterochromatic blocks on all chromosomes and a terminal heterochromatic region on the *q* arm of the 2^nd^ pair. Mapping 18S rDNA gave hybridization signals, correlated with the nucleolus organizer regions, on the 2^nd^ pair for all analyzed individuals. However, we found distinct patterns for 5S rDNA: interstitially at the proximal position on 6^th^ pair of four species (*C. kelberi*, *C. pinima*, *C. piquiti* and *C. vazzoleri*), and on the distal of the 4^th^ pair in two (*C. monoculus* and *C. temensis*). Accordingly, we present here new data for the genus and discuss the evolutionary trends in the karyotype of this group of fish. In addition, we provide data that supports the occurrence of hybrid individuals in the Uatumã River region, mainly based on 5S rDNA mapping.

## Introduction

The genus *Cichla* Bloch et Schneider, 1801 belongs to the subfamily Cichlinae that, jointly with *Retroculus* Eigenmann et Bray, 1894, makes up the tribe Cichlini, and is the earliest-diverging lineage of Neotropical cichlids (Leo Smith et al. 2008). This taxon is widely distributed within the Amazon, Tocantins, and Orinoco River basins, and in the smaller rivers draining the Guianas to the Atlantic Ocean. Most *Cichla* species follow an allopatric distribution pattern, although some species are sympatric or even syntopic (Kullander and Ferreira 2006). However, some species, such as *C. monoculus* Agassiz, 1831, *C. kelberi* Kullander et Ferreira, 2006 and *C. piquiti* Kullander et Ferreira, 2006, have been introduced into other areas, where they are well established, due to their generalist habit. *Cichla* are very emblematic fish in South America, with high economic and ecological importance, especially since they are predators in Amazonian rivers and used widely for sport fishing (Nascimento et al. 2001; dos Santos et al. 2016; Diamante et al. 2017).

Representatives of the genus *Cichla* are easily distinguished from all other Neotropical cichlids by the shape of the dorsal fin, and the presence of 1 to 4 dark vertical bars along the body. However, the species are very similar, and while their color patterns still provide the best species diagnostic characters, in some cases these may complicate accurate identification, since key characters may show ontogenetic changes (Kullander and Ferreira 2006).

According to Kullander and Ferreira (2006), the genus comprises 15 morphologically distinct species, and recently another species has been described (*Cichla cataractae*Sabaj et al. 2020) from the Essequibo River basin, where it is endemic). However, Willis et al. (2012), based on multilocus data, recognized only eight species. The species often have restricted natural distributions, but to variable extents. For example, while *C. monoculus* is found all over the Amazon River and low tributary course, *C. temensis* Humboldt, 1821 is found only in black water rivers, whereas *C. piquiti* and *C. kelberi* are restricted to the Tocantins River.

Cytogenetic data concerning the family Cichlidae points to a remarkable trend in the maintenance of the diploid number 2n = 48, mostly in the acrocentric form (Thompson 1979). However, as more species were karyotyped, a huge chromosomal diversity was observed in the derived clades (ranging from 32 to 60 chromosomes), but with predominance of 2n = 48 in most lineages, which is considered an ancestral trait for this group (Feldberg et al. 2003; Gross et al. 2009; Poletto et al. 2010; da Costa et al. 2019). For the genus *Cichla*, only *C. monoculus*, *C. temensis*, *C. kelberi* and *C. piquiti* have had their karyotypes described, all exhibiting a diploid number composed of 48 acrocentric chromosomes, as the species from earliest-diverging Cichlinae tribes (Retroculini, Astronotini and Chaetrobranchini) (Feldberg et al. 2003; Alves-Brinn et al. 2004; Poletto et al. 2010; Mourão et al. 2017).

Interestingly, Alves-Brinn et al. (2004), based on cytogenetic data, reported the occurrence of hybridization between *C. monoculus* and *C. temensis* in the Uatumã River (Balbina Hydroelectric Dam). In addition, interspecific hybridization and introgression between species has been much discussed in relation to the adaptive advantages and increase of genetic variability (Willis et al. 2012). For some authors, hybridization may be related to diversification and speciation, or the extinction of populations or species (Mourão et al. 2017). Under either species concept, the phylogenetic breadth of introgression in this group is clear, with both sister species and species from different mtDNA clades exhibiting genetic introgression (Willis et al. 2012).

In the current study, we used different classical and molecular cytogenetic markers to characterize *Cichla* species, from different river drainages within the Amazon basin and investigate the likely existence of hybrid individuals, where more than one species occurs, such as at Uatumã River (Balbina Hydroelectric Dam).

## Material and methods

**Figure 1.**
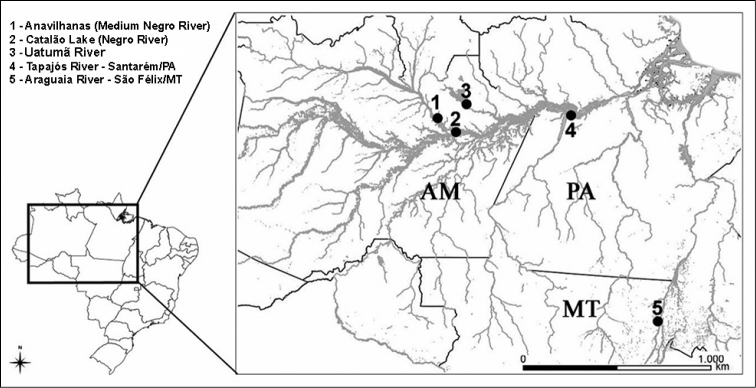
Map showing the collection points of *Cichla* species analyzed in current study.

**Figure 2.**
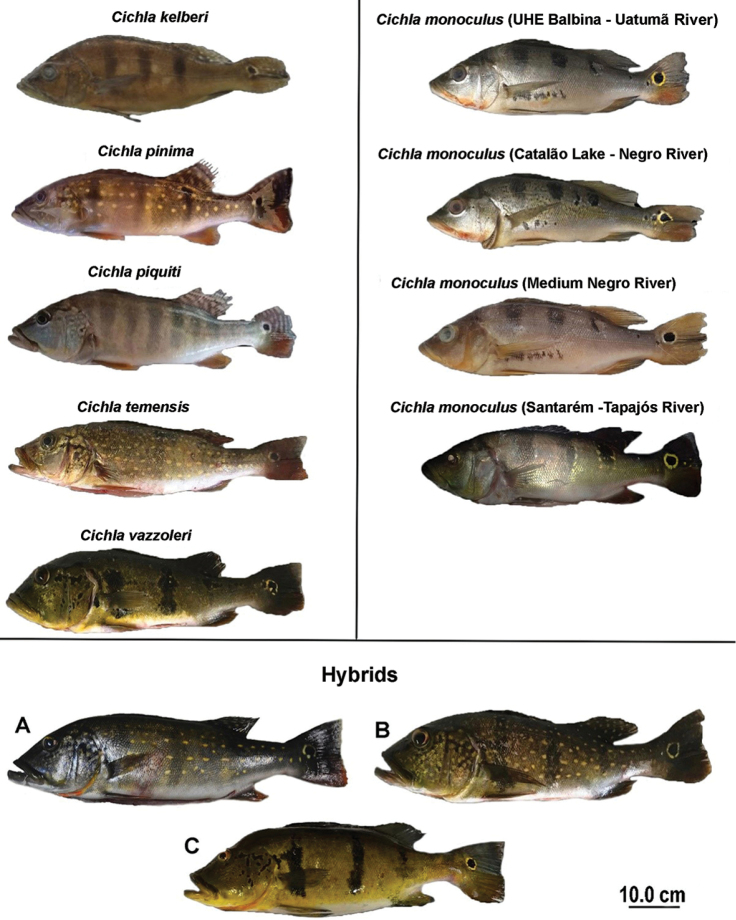
*Cichla* species and individuals considered morphologically hybrid (**A–C**). *C. kelberi* SL = 170.0 mm; *C. pinima* SL = 190.5 mm; *C. piquiti* SL = 200.0 mm; *C. temensis* SL = 210.0 mm; *C. vazzoleri* SL = 250.0 mm; *C. monoculus* (Uatumã River) SL = 160.0 mm; *C. monoculus* (Catalão Lake, Negro River) SL = 150.0 mm; *C. monoculus* (Anavilhanas, Medium Negro River) SL = 180.0 mm; *C. monoculus* (Santarém, Tapajós River) SL = 180.0 mm; Hybrid **A** SL = 280.0 mm; Hybrid **B** SL = 230.0 mm; Hybrid **C** SL = 320.0 mm.

In the current study, we sampled 50 individuals of the genus *Cichla* from five locations in the Brazilian Amazon basin (Table 1, Figs 1, 2) under ICMBIO (Instituto Chico Mendes de Conservação da Biodiversidade) permit number: 28095-1. Voucher specimens were deposited in the Fish Collection of the National Institute of Amazonian Research (Instituto Nacional de Pesquisas da Amazônia – INPA) (Table 1). Dr. Efrem Ferreira and Dr. Jansen Zuanon, following description of Kullander and Ferreira (2006), identified the *Cichla* species included in the current study. However, three individuals had mixed characteristics of more than one species, and were thus considered possible hybrids by specialists.

**Table 1.** The *Cichla* species included in the current study, collecting localities, the number of individuals analyzed, and Voucher number. ♂ = male, ♀ = female. AM = Amazonas State, PA = Pará State, MT = Mato Grosso State.

Species	Number of individuals	Collecting localities	Coordinates	Voucher
*C. kelberi*	4♂ 3♀	Araguaia River – São Félix, MT	11°39'03.9"S, 50°52'59.4"W	MZUSP125273
*C. monoculus*	5♀	Anavilhanas (Negro River), AM (Black water)	2°33'28.4"S, 60°46'29.7"W	INPA-ICT059045
*C. monoculus*	3♀	Uatumã River (Balbina Hydroelectric Dam) AM, Black water)	1°55'02.2"S, 59°28'23.7"W	INPA-ICT059046
*C. monoculus*	1♀	Tapajós River – Santarém, PA (Clear water)	2°24'53.0"S, 54°46'48.3"W	INPA-ICT059047
*C. monoculus*	4♂ 1♀	Catalão Lake, AM (Mix of white and black water)	3°10'30.8"S, 59°56'30.3"W	INPA-ICT059044
*C. pinima*	7♂ 6♀	Tapajós River (Mix of white and clear water)	24°21'16.4"S, 54°70'23.16"W	INPA-ICT059045
*C. piquiti*	2♂ 2♀	Araguaia River – São Félix, MT	11°38'01.7"S, 50°40'11.3"W	MZUSP125272
*C. temensis*	2♂ 2♀	Uatumã River (Balbina Hydroelectric Dam). AM, Black water)	1°55'02.2"S, 59°28'23.7"W	INPA-ICT059043
*C. vazzoleri*	2♂ 3♀	Uatumã River (Balbina Hydroelectric Dam, AM, Black water)	1°55'02.2"S, 59°28'23.7"W	INPA-ICT059048
Hybrids	3♂	Uatumã River (Balbina Hydroelectric Dam, AM, Black water)	1°55'02.2"S, 59°28'23.7"W	INPA-CT059047

Chromosomal preparations were obtained from the kidney, following the protocol of Gold et al. (1990). The active nucleolus-organizing region (NOR) was detected with silver nitrate impregnation (Ag-NOR), following Howell and Black (1980), while constitutive heterochromatin was detected following Sumner (1972). DNA was extracted using the Wizard Extraction Kit (Promega), following manufacturer’s recommendations, and quantified using a NanoVue Plus spectrophotometer (GE Healthcare).

Amplification of 18S and 5S rDNA used the Polymerase Chain Reaction (PCR) with primers 18S F(5’ -CCG CTT TGG TGA CTC TTG AT-3’) and R(5’ -CCG AGG ACC TCA CTA AAC CA-3’) (Gross et al. 2010), and the primers 5S F(5’ -TAC GCC CGA TCT CGT CCG ATC-3’) and R(5’ -CAG GCT GGT ATG GCC GTA AGC-3’) (Martins and Galetti 1999).

All PCRs were performed with a final volume of 25 μL, containing genomic DNA of each species (200 ng), 10× buffer with 1.5 mM MgCl_2_, DNA polymerase (5 U/μL), dNTPs (1 mM), primers (5 mM) and Milli-Q. The reaction profile for 18S rDNA was 1 min. at 95 °C, 35 cycles of 1 min. at 94 °C, 1 min. at 56 °C and 1 min. and 30 s at 72 °C, followed by 5 min. at 72 °C. The reaction profile for 5S rDNA amplification was 1 min. at 95 °C, followed by 30 cycles of 1 min. at 94 °C, 1 min. at 59 °C and 1 min. and 30 s at 72 °C. The final extension was 5 min. at 72 °C. PCR products were checked on 1% agarose gel, quantified on a NanoVue Plus spectrophotometer (GE Healthcare). PCR products were labeled with digoxigenin (Dig-Nick Translation mix; Roche) and biotin (Bio-Nick Translation mix; Roche), and used as probes for the fluorescent *in situ* hybridization technique (FISH).

Hybridizations were performed according to the protocol described by Pinkel et al. (1986), with a stringency of 77% (2.5 ng/μL) for 18S rDNA, 5S rDNAr, 50% formamide, 10% dextran sulfate and 2xSSC at 37 °C for 18 h), post-hibridization washes were made with formamide 15% and 2xSSC Tween 0.5%. Chromosomes were counterstained with DAPI (2 mg/mL) using the Vectashield (Vetor) mounting medium. Telomeric segments were generated using non-templated PCR with *primers* (TTAGGG)5 and (CCCTAA)5 (Ijdo et al. 1991).

We analyzed at least 30 metaphase per individual to confirm the diploid number and karyotype structure. Images were captured using an Olympus BX51 epifluorescence microscope, and processed using Image-PRO MC 6.0 softwares. Chromosomes were measured using the Image J program, arranged in descending order of chromosome size, and classified according to Levan et al. (1964).

All methodological procedures in the current study were performed in accordance with the guidelines of the Ethics Committee of the National Institute of Amazonian Research (Instituto Nacional de Pesquisas da Amazônia – INPA), protocol: CEUA No. 009/2018.

## Results

**Figure 3.**
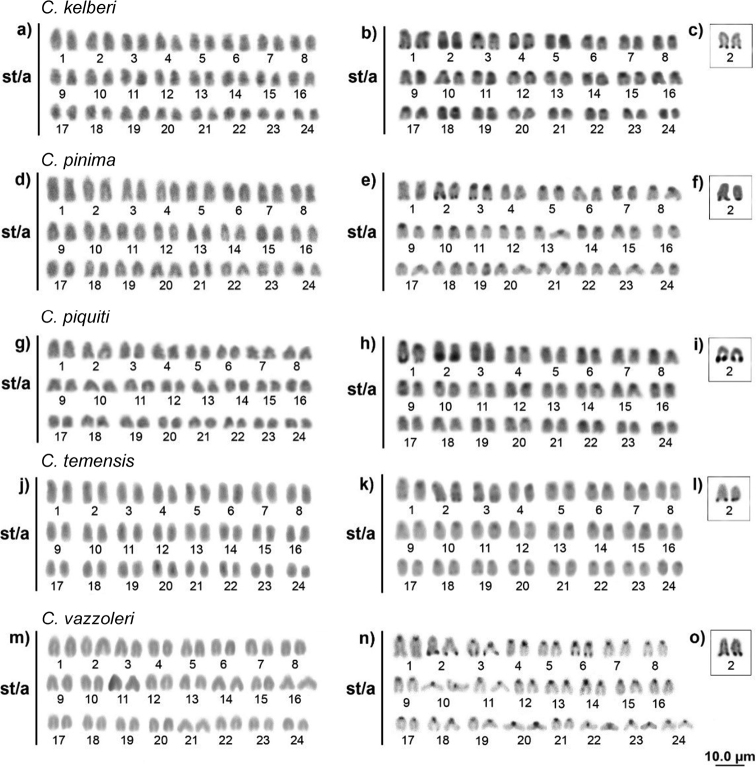
Karyotypes analyzed by conventional Giemsa staining, C banding and Ag-NOR: *Cichla kelberi* (**a, b, c**) *C. pinima* (**d, e, f**) *C. piquiti* (**g, h, i**) *C. temensis* (**j, k, 1**) *C. vazzoleri* (**m, n, o**).

**Figure 4.**
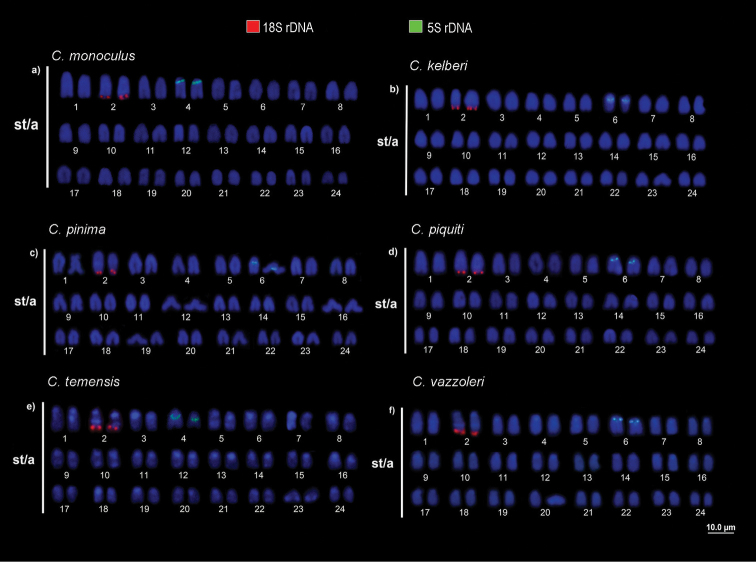
Karyotypes analyzed with molecular chromosome markers. Double FISH with 18S (red) and 5S (green) rDNA probes. *Cichla monoculus* (**a**) *C. kelberi* (**b**) *C. pinima* (**c**) *C. piquiti* (**d**) *C. temensis* (**e**) *C. vazzoleri* (**f**).

The six species analyzed (*C. kelberi*, *C. monoculus*, *C. pinima*, *C. piquiti*, *C. temensis* and *C. vazzoleri*) all had a diploid number equal to 48 acrocentric chromosomes, and a fundamental number (FN) equal to 48. The NORs (Ag-NORs and 18S rDNA) were located in a distal position on the *q* arms of pair n° 2 in all species (Figs 3, 4). *Cichla monoculus* was the sole species sampled in more than one location, and it showed no difference when compared to data in Alves-Brinn et al. (2004) and Schneider et al. (2013) (data not shown). The 5S rDNA site was located interstitially at the proximal position of pair n° 6 in four species (*C. kelberi*, *C. pinima*, *C. piquiti* and *C. vazzoleri*), and on distal portion of pair n° 4 in two (*C. monoculus* and *C. temensis*) (Fig. 4).

**Figure 5.**
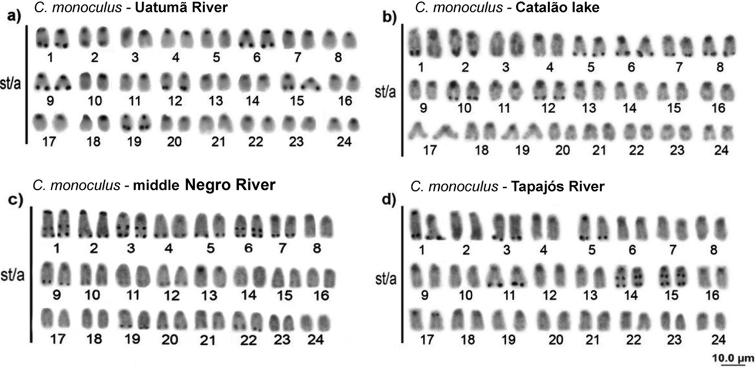
*Cichla monoculus* karyotype from different locations with conventional Giemsa staining, C. banding: **a** Uatumã River **b** Catalão Lake (Negro River) **c** Anavilhanas (middle Negro River) **d** Tapajós River.

The six species had centromeric heterochromatic blocks on all chromosomes and a terminal heterochromatic region on pair 2, which corresponds to the same position as the NORs. However, some blocks were species-specific: terminal blocks were observed on the *q* arm of *C. kelberi* pairs 1, 3 and 4; pair 3 of *C. pinima* and *C. temensis*; pairs 1 and 3 of *C. piquiti*; and in *C. vazzoleri* pairs 3 and 6 (Fig. 3). *C. monoculus*, which was sampled in four different locations, showed variable constitutive heterochromatin patterning, where individuals from the Uatumã River (Balbina Hydroelectric Dam) also had terminal blocks on the *q* arms of the chromosomal pairs 1, 6, 9, 12, 15, 19. Catalão Lake individuals appeared to have terminal pale blocks on all pairs, with conspicuous ones on 1, 5, 6, 8, 10 chromosomal pairs. This also occurred for individuals from Anavilhanas, but in these, the blocks were more conspicuous in practically all chromosomes, and still had interstitial markings on pairs 1, 3 and 6. Individuals from the Tapajós River had terminal blocks on pairs 1, 3, 5, 11, 14, 15, and interstitials on pairs 14 and 15 (Fig. 5).

**Figure 6.**
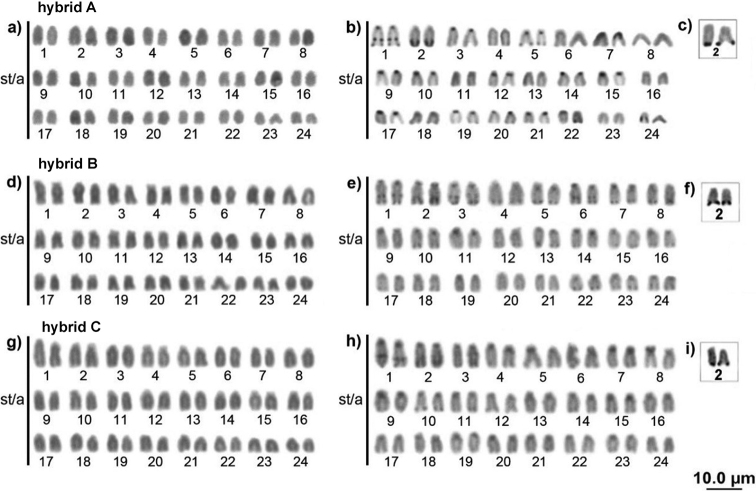
Karyotype of hybrid individuals A, B and C (as in Fig. 2) respectively, with conventional Giemsa staining (**a, d, g**), C-banding (**b, e, h**) and Ag-NOR (**c, f, i**).

**Figure 7.**
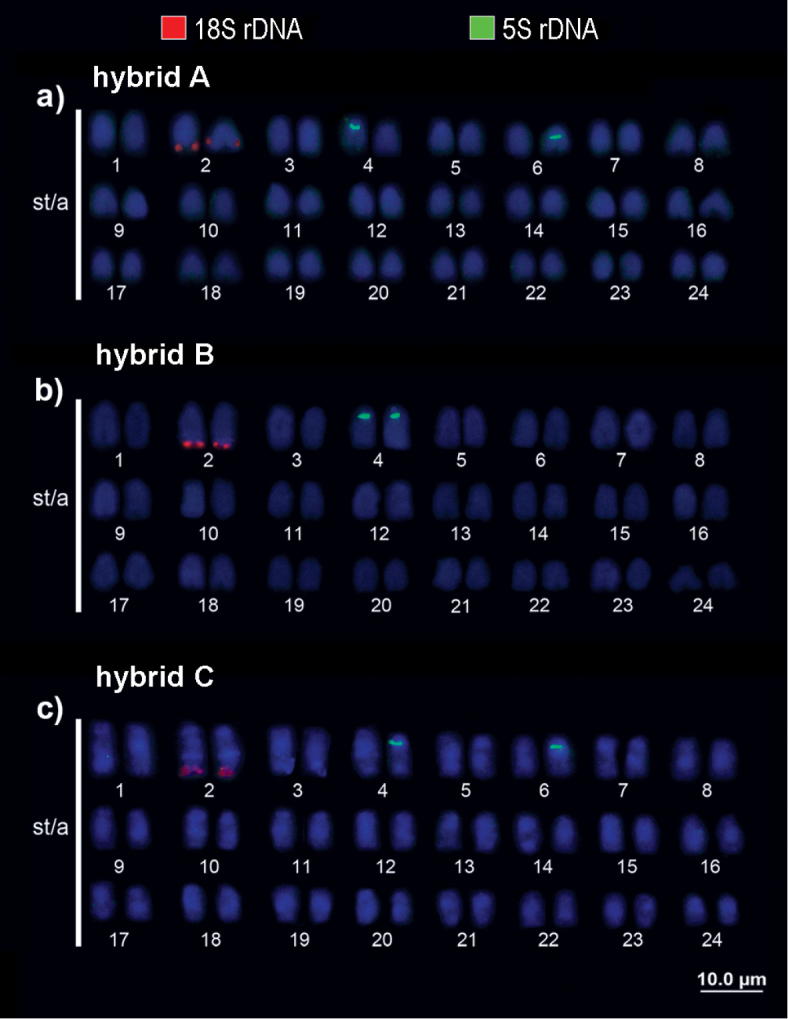
Karyotypes of hybrid individuals **A**, **B** and **C** (as shown in Fig. 2) respectively with molecular chromosomal markers. Double FISH with 18S (red) and 5S (green) rDNA probes.

The three individuals morphologically considered hybrids also had 2n = 48 acrocentric chromosomes and FN = 48, Ag-NOR and 18S rDNA on the second pair at terminal position on the *q* arm, collocated with a conspicuous heterochromatic portion (Figs 6, 7). Constitutive heterochromatin was present in the centromeric region of all chromosomes in the three individuals and the first pair had an interstitial block. Additionally, individual A (Fig. 6b) had terminal blocks on pairs 1 and 5; individual B (Fig. 6e) had terminal blocks on most chromosomes and interstitials on pairs 3 and 6; individual C (Fig. 6h) had terminal pale blocks on pairs 3 and 10.

5S rDNA was detected interstitially on one pair 4 homolog, and on one pair 6 homolog in two hybrid individuals (A and C). Individual B showed 5S rDNA sites on both pair 4 chromosomes (Fig. 7).

For all analysed species, hybridization with telomeric probes showed, as expected, only markings on the terminal portions of both arms (data not shown).

## Discussion

For Cichlidae species, a diploid number equal to 48 acrocentric-like chromosomes is considered an ancestral feature (Thompson 1979), and chromosomal evolution in this family was thought to be conserved from the karyotype macrostructure point of view (Feldberg et al. 2003). However, as more Cichlidae species were cytogenetically studied and more accurate techniques were applied (e.g. mapping of different molecular chromosomal markers), several karyotypic formulas and configurations have been found (Gross et al. 2009; Poletto et al. 2010; Schneider et al. 2013), suggesting that this fish group experienced multiple non-robertsonian chromosomal rearrangements during its evolution, since the 2n = 48 is retained in most Cichlinae lineages.

In the current study, analyzes focused on the genus *Cichla*, which represents one of the most basal lineages of Neotropical cichlids (Leo Smith et al. 2008). To date, all species karyotyped possess a complement of 48 acrocentric-like chromosomes, with very similar karyotypes between species, including the NOR pattern, which is usually found on the 2^nd^ pair (Alves Brinn et al. 2004; Schneider et al. 2013; Mourão et al. 2017; current study). In addition, some studies examining morphological-mitochondrial divergences (Andrade et al. 2001; Willis et al. 2010), as well as chromosome features (Alves Brinn et al. 2004; Oliveira et al. 2006), and electrophoretic esterase comparisons (Teixeira and Oliveira 2005) have inferred hybridization in natural and in artificial or disturbed environments/populations.

For constitutive heterochromatin, the distribution pattern can often be used as a species-specific or population marker (Feldberg et al. 2003; Vicari et al. 2006; Benzaquem et al. 2008; Perazzo et al. 2011). In our analyses, for instance, we found four different heterochromatin patterns for *C. monoculus* (Fig. 5). This is one of the most widely-distributed species in the Amazon River basins (Kullander and Ferreira 2006), and the commonly introduced into dam resevoirs throughout Brazil (dos Santos et al. 2016; Diamante et al. 2017). Heterochromatin is known to play important roles in the chromosomal architecture and karyotype organization, such as assisting in chromosomal segregation, nuclear organization and expression of gene regulation, associated with responses to environmental changes (Grewal and Jia 2007; Varriale et al. 2008; Bühler 2009; Ribeiro et al. 2017; Viana Ferreira et al. 2019). This seems to be the case for the different *C. monoculus* populations analyzed in our study, where individuals from black and acid waters (Negro and Uatumã rivers), white and black mixed waters (confluence of Negro and Solimões rivers), and in the confluence of white and clear waters (confluence of Amazonas and Tapajós rivers), showed intraspecific variability in their heterochromatic patterns, possibly reflecting chromatin adaptation and/or epigenomic responses to changes in the specific environment inhabited by these different populations.

Interestingly, the heterochromatic patterns of the three probable hybrids was very similar and much closer to the pattern described for the Negro River (*C. monoculus* from Anavilhanas) with some interstitial blocks. Could it be heterochromatinization? Such heterochromatin variability can also be explained by stressors, such as environmental changes, or even hybridization processes (Richards et al. 2010; Ribeiro et al. 2017), which would explain the heterochromatin distribution differences found in *C. monoculus* and in the probable hybrids.

Besides the conservation of the karyotype macrostructure, Schneider et al. (2013) reported that 12 out of 13 Cichlinae species analyzed in their study had only one chromosome pair harboring 5S rDNA sites, but at different karyotypic positions, indicating that 5S rDNA sites are a robust molecular chromosomal marker in cichlid species. 5S rDNA is an important cytotaxonomic and evolutionary marker, since it helps provide a better understanding of fish chromosomal diversity (Bellafronte et al. 2005; Teixeira et al. 2009; Vicari et al. 2010). For instance, a study by Ferreira et al. (2016), mapping of 5S rDNA sequences in *Bunocephalus coracoideus* Cope, 1874, revealed an association between this rDNA site and a multiple sex chromosome system previously unknown in Siluriformes (X_1_X_1_X_2_X_2_/X_1_Y_1_X_2_Y_2_). Repetitive 5S and 18S rDNA sequences are the most well-studied in fish, and have been gaining prominence mainly in studies of between-species evolutionary relationships, population characterization and genome structure (Martins et al. 2004; Terencio et al. 2012; Schneider et al. 2013).

In the current study, individuals of all six species, including the hybrids, had 18S rDNA on terminal position of the *q* arm of the 2^nd^ chromosomal pair (same position as NORs). Meanwhile, 5S rDNA mapping in *Cichla* species showed two patterns: on the 4^th^ pair (*C. monoculus* and *C. temensis*), and on the 6^th^ pair (*C. kelberi*, *C. pinima*, *C. piquiti* and *C. vazzoleri*). However, in the individuals morphologically considered hybrids, we found two distinct patterns: two of them (hybrids A and C) having 5S rDNA in one homologue of the 4^th^ pair and one homologue of the 6^th^ pair, while the other hybrid (individual B) had 5S rDNA on both homologues of the 4^th^ pair. Since these probable hybrids were captured in the Uatumã River (Balbina Hydroelectric Dam), where *C. monoculus*, *C. temensis* and *C. vazzoleri* all occur (Kullander and Ferreira 2006), we believe that these species might be hybridizing.

Interestingly, the karyotypes of *C. pinima* and *C. vazzoleri* (current study) are very similar, except for a heterochromatic terminal block on the *q* arms of the 6^th^ pair in *C. vazzoleri*. It is notable that *C. pinima* was sampled in the Tapajós River and *C. vazzoleri* in the Uatumã River, very distant locations with no history of sympatry or migration (Ferreira, personal communication). However, according to Willis (2017), *C. pinimasensu lato* includes *C. pinima*, *C. vazzoleri*, *C. jariina*, Kullander et Ferreira, 2006 and *C. thyrorus* Kullander et Ferreira, 2006 (*sensu*Kullander and Ferreira 2006), and reports that the evolutionary relationships in this group are more complex than previously thought. Willis (2017) suggest that this separation into four species does not correspond to its evolutionary history and contemporary dynamics of the genus.

In addition, Willis et al. (2012) reported that genetic introgression is a common phenomenon in *Cichla* species. Introgression can be defined as the movement of DNA from the genetic pool of one species into that of another species by repeated backcrossing of hybrid individuals with one or both parent species. Such hybridization events are expected to occur most commonly in modified habitats, but interestingly, most of the hybridization cases known for *Cichla* species, were found in undisturbed natural environments (Willis et al. 2012), suggesting that introgression forms a natural part of the evolution of many tropical species, so increasing genetic diversity. In this sense, we cannot rule out hybridization and genetic introgression among the likely parental species, especially taking in account that all three probable hybrid individuals used here had male gonads.

## Conclusions

Our data supports the tendency in the maintenance of the 2n = 48 chromosomes for *Cichla* species, as well as the conservation of the karyotypic formula and simple NOR, but reveals 5S rDNA to be an important cytogenetic marker for this group. In additon, here we provide, for the first time, the karyotype for *C. pinima* and *C. vazzoleri*. Furthermore, our data shows that the heterochromatin pattern may differentiate populations of *C. monoculus*, suggesting that this variation might be the result of epigenetic events triggered by different water types.
